# Molecular Characterization of *Cryptosporidium* spp. in Brandt's Vole in China

**DOI:** 10.3389/fvets.2020.00300

**Published:** 2020-06-30

**Authors:** Shengyong Feng, Han Chang, Ye Wang, Chengmei Huang, Shuyi Han, Hongxuan He

**Affiliations:** ^1^National Research Center for Wildlife Borne Diseases, Institute of Zoology, Chinese Academy of Sciences, Beijing, China; ^2^College of Life Sciences, University of Chinese Academy of Sciences, Beijing, China

**Keywords:** prevalence, public health, zoonoses, phylogenetic analysis, genotyping

## Abstract

*Cryptosporidium* spp. are important intestinal parasites that infect humans and various animals, including wildlife. Currently, few epidemiological data in wild rodents, especially in voles, are available. In the present study, a total of 678 Brandt's vole feces samples were collected from Maodeng Livestock Farm and East Ujimqin, Inner Mongolia. The overall prevalence of *Cryptosporidium* spp. was 18.7%. Significant differences were not found between genders but between locations and weight groups. Moreover, three known species/genotypes, *C. suis, Cryptosporidium* environmental sequence and muskrat genotype II, and a novel *Cryptosporidium* species/genotypes of Brandt's vole was identified. To the best of our knowledge, this is the first report of *Cryptosporidium* spp. infection in Brandt's vole worldwide. These findings imply Brandt's voles might be a potential source of human cryptosporidiosis.

## Introduction

Cryptosporidiosis, caused by species of the genus *Cryptosporidium*, is one of the common etiologies of diarrhea in humans and animals (1). The oocysts shed from infected hosts can survive for quite a long time in the environment (2). Thus, infection is more likely to occur by ingesting water or foods contaminated with oocysts (3). The prognosis of cryptosporidiosis may be chronic infection or life-threatening in certain people (4).

Rodents can carry a large number of pathogens, including bacteria, viruses and parasites, which pose a threat to public health (5). Brandt's vole (*Lasiopodomys brandtii*) is a small, non-hibernating, herbivorous rodent species, and mainly distributed in the grasslands of Inner Mongolian of China, Mongolia, and Southeast Baikal region of Russia (6). It is generally agreed that Brandt's vole is one of the important grassland pests due to their damage to grasslands (6).

Currently, *Cryptosporidium* has been identified from domestic mammals (7), birds (8), reptiles (9), amphibians (10), and fishes (11). More than 40 species of *Cryptosporidium* have been identified (7). However, it is rarely reported in wild rodents, especially in voles. Here we reported the prevalence of *Cryptosporidium* spp. in wild Brandt's vole from Inner Mongolian, China. Data from this study contributes to enriching the epidemiological data of *Cryptosporidium* in China.

## Materials and Methods

### Ethics Statement

This study was conducted in accordance with the Guidelines for the Care and Use of Animals in Research, which are issued by the Institute of Zoology, Chinese Academy of Sciences. This work was reviewed and approved by the Animal Ethics Committee of the Institute of Zoology, Chinese Academy of Sciences.

### Sample Collection and DNA Extraction

From 2017 to 2018 (August to September each year), Brandt's voles were trapped using live traps baited with peanuts (12) at two discontinuous habitats, Maodeng Livestock Farm (MD) and East Ujimqin (DWQ), of Xilingol Grassland, Inner Mongolia, China. The climatic conditions between DWQ and MD are similar. However, MD is experiencing more anthropogenic disturbances, such as village and grazing activity, compared with DWQ (13). Sampling was conducted before 10 a.m. and after 5 p.m. Fecal samples were collected into 2 ml sterilized centrifuge tubes from each trap. The sex, weight and reproductive status of the captured Brandt' voles were recorded. The trapped vole was released after recording the individual details. The tubes were marked, put into a box filled with ice packs and transported to a refrigerator as soon as possible.

The total genomic DNA was extracted from 200 mg feces with the EZNA® Stool DNA Kit (Omega Biotek Inc., Norcross, USA) following the manufacturer's instructions. The purified DNA was stored at −20°C for further PCR. *Cryptosporidium* species/genotypes were determined by amplifying the SSU rRNA gene under nested PCR according to the previous studies (14, 15). Positive control and negative control are added in each amplification. The secondary PCR products were visualized by 2% agarose gel electrophoresis containing GoldView™ (Solarbio, China) stained.

### Sequencing and Phylogenetic Analyses

Positive secondary PCR products were bi-directionally sequenced by the Sino Geno Max Company (Beijing, China). Chromatograms of the forward and reverse sequences were manually confirmed and assembled with Lasergene SeqMan software (DNASTAR, Madison, Wisconsin, USA). Cryptosporidium species/genotypes were determined by aligning with reference sequences available in GenBank database with the ClustalX 1.83 software package. Phylogenetic relationship of Cryptosporidium spp. was constructed under MEGA 7.0 (16) with the Neighbor-joining algorithm in Jukes-Cantor method (17), and the robustness of clusters was estimated using a bootstrap of 1, 000 replicates (18).

### Statistical Analysis

Differences in infection rates were compared with the chi-square test under SPSS 19.0 (SPSS Inc., Chicago, USA). Differences were considered to be statistically significant when *P* < 0.05.

## Results

### Prevalence of *Cryptosporidium* Spp. in Brandt's Voles

In total, 678 Brandt's voles were sampled from DWQ and MD of Inner Mongolia Autonomous Region, China. 127 samples (18.7%) were found to be *Cryptosporidium*-positive by testing the partial small subunit (SSU) rRNA gene via PCR. The infection rates of *Cryptosporidium* spp. in these two regions were 15.6 and 23.6% (χ^2^ = 6.845, *df* = 1, *P* = 0.009), respectively. The prevalence of *Cryptosporidium* spp. in female Brandt's voles (18.9%) was quite similar to that in male Brandt's voles (18.5%) (χ^2^ = 0.018, *df* = 1, *P* = 0.893). The infection rate of voles weighing <25 g was significantly higher than those weighing between 25 and 35 g and those weighing more than 35 g (χ^2^ = 17.753, *df* = 2, *P* = 0.000) (Table 1).

**Table 1:** Prevalence and distribution of *Cryptosporidium* species/genotypes in Brandt' voles in Inner Mongolia, China.

**Factors**	**Category**	**Prevalence**	**(95%CI)**	***P*-value**
		**(No. positive/No. tested)**		
Gender	Female	18.9% (61/322)	14.64–23.25	0.893
	Male	18.5% (66/356)	14.48–22.60	
Weight	≤25	30.2% (45/149)	22.74–37.66	0.000
	25–35	16.9% (59/350)	12.92–20.80	
	>35	12.8% (23/179)	7.89–17.80	
Location	DWQ	15.6% (64/411)	1205–19.09	0.009
	MD	23.6% (63/267)	18.47–28.72	
Total		18.7% (127/678)	15.79–21.68	

### *Cryptosporidium* Species/Genotypes

**Figure 1:**
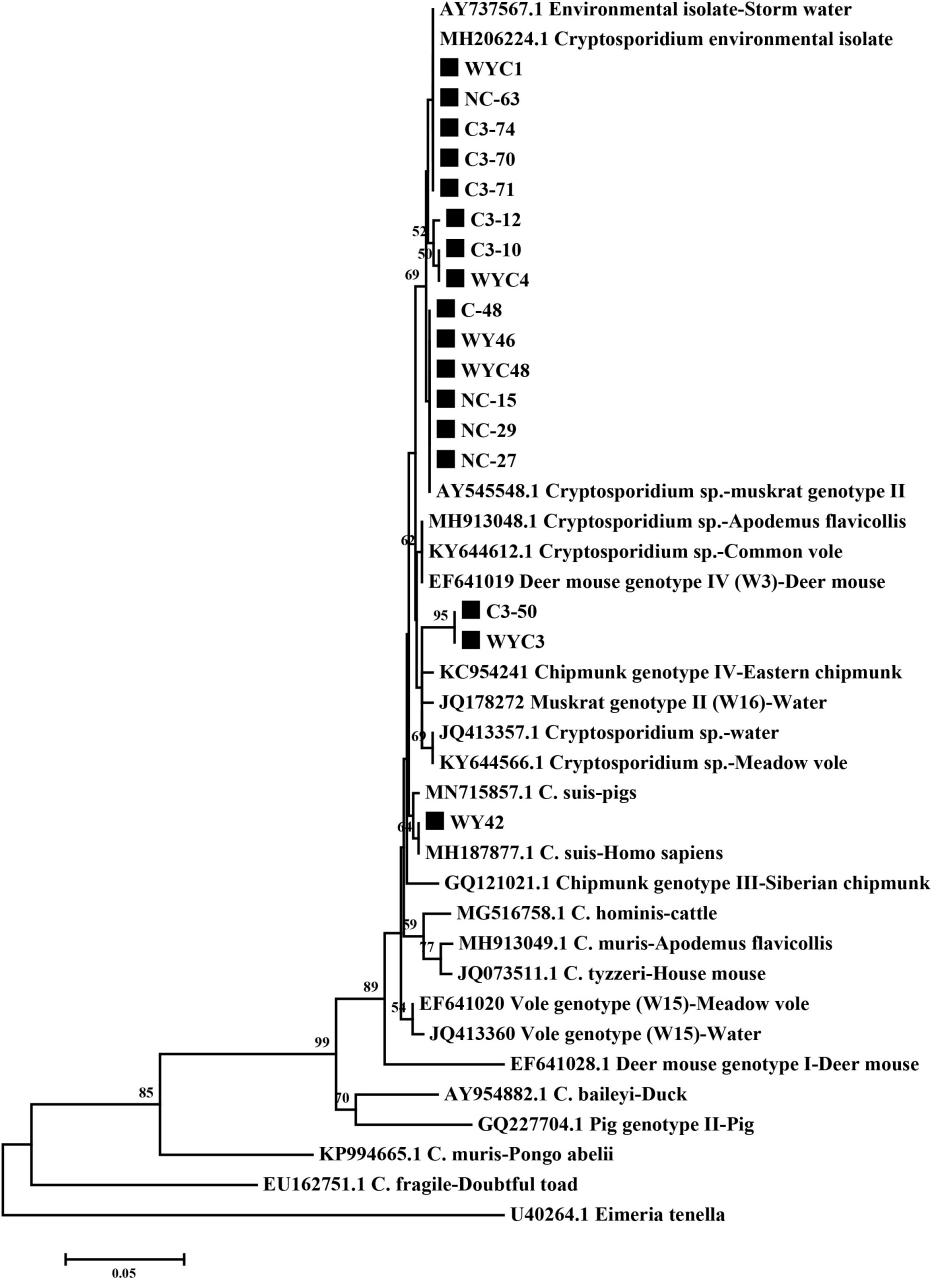
Phylogenetic analysis of *Cryptosporidium* spp. using Neighbor-Joining (NJ) method based on sequences of the small subunit ribosomal RNA (SSU rRNA) gene. Bootstrap values >50% are shown (1,000 replicates). Isolates obtained in the present study are indicated by solid square. The SSU rRNA gene sequence of *Eimeria tenella* is used as the outgroup.

Of the 127 PCR positive samples, 122 were sequenced successfully. Furthermore, 17 representative sequences were obtained through sequence analysis. Four *Cryptosporidium* species/genotypes of Brandt's voles were identified by aligning against the reference *Cryptosporidium* sequences and constructing phylogenetic tree with the SSU rRNA gene sequences (Figure 1), including three known species, C. suis, Cryptosporidium environmental sequence and Cryptosporidium muskrat genotype II, and one novel *Cryptosporidium* genotypes, termed *Cryptosporidium* Brandt's voles genotype I (Figure 1). The Brandt's voles genotype I showed significant differences from other known *Cryptosporidium* spp. or genotypes in the SSU rRNA sequences. Except for the isolate WY42 is identical with the known sequence (MH187877, *C. suis*), sequence heterogeneity was observed in other two known *Cryptosporidium* species/genotypes. The sequences clustered with *Cryptosporidium* muskrat genotype II exhibit two nucleotide insertions (A at position 461 and T at position 469). Three types of sequences were seen in *Cryptosporidium* environmental sequences with some substitutions (Table 2).

**Table 2:** Variations in the SSU nucleotide sequences among *Cryptosporidium* environmental sequences in the present study.

**Sequence types**	**Nucleotide at position**					
	**425**	**465**	**466**	**468**	**469**	**621**
Reference (AY737567)	C	T	A	T	A	T
Type I	C	T	T	T	A	T
Type II	T	A	T	A	T	C
Type III	C	T	T	T	A	C

## Discussion

*Cryptosporidium* spp. is one of an important apicomplexan parasite. Many studies have shown that *Cryptosporidium* spp. can infect humans and animals, and many *Cryptosporidium* species/genotypes exhibiting public health significance have been found (7). However, it is rarely reported in rodents, especially in voles (19, 20). In this study, we first characterized the prevalence of *Cryptosporidium* spp. in Brandt's voles.

The prevalence of *Cryptosporidium* spp. infection varies with species and sampling locations.

In the present study, the overall prevalence of *Cryptosporidium* spp. in Brandt's voles was 18.7%, which was higher than that in Qinghai voles (8.9%, 8/90) from China (21), common voles (14.2%, 50/353) and bank voles (7.1%, 10/140) from Europe (22), and lower than that in common voles (22.6%, 74/328) from Czech Republic (23), in common voles (73%, 200/274) from Poland (24), and in meadow voles (52.4%; 163/311) from USA (22). The sample size may also be the causation of the difference in prevalence. Furthermore, the prevalence difference between female and male Brandt's voles was not significant, but there were significant differences in body weight and sampling location, respectively. To a certain degree, Rodent's weight can represent its age. The present study showed that prevalence of *Cryptosporidium* spp. in Brandt's voles was negatively correlated with age, and the youngest voles were significantly higher than the other two groups, which is consistent with previous reports (25). Stronger immunity in older Brandt's voles may lower the infection rates. Both DWQ and MD have similar climatic conditions, while MD is experiencing more anthropogenic disturbances, such as village and grazing activity, compared with DWQ, which may contribute to the difference of parasite prevalence (26).

*C. suis*, a zoonotic potential species of *Cryptosporidium*, are commonly detected in pigs (27–29). Other host, such as *Cervus unicolor* (Reference not published, access number: KX668209), *Vulpes vulpes* (Reference not published, access number: MN996816), and *Apodemus flavicollis* (20), were also found to be infected by the parasite. As far as we know, this species was first reported in Brandt's Vole, which suggests that Brandt's Vole might be a potential source of human cryptosporidiosis. Other two known species/genotypes, Cryptosporidium environmental sequence and Cryptosporidium muskrat genotype II, have been found in other environmental samples (30–32), which suggests that environment plays an important role in transmission dynamics of the parasites. Future studies to characterize the prevalence of the parasites in environmental samples from the grassland areas is needed.

Moreover, several loci differences exist in the sequences of Cryptosporidium environmental sequence and Cryptosporidium muskrat genotype II which are in line with previous studies that the heterogeneity of *Cryptosporidium* SSU sequence was higher (22). Previous studies have shown that the host range of *Cryptosporidium* genotypes found in arvicolinae is relatively limited (e.g., *Cryptosporidium* muskrat II were commonly detected in voles than other hosts), which may be the result of host divergence (22). Whether these novel genotype found in this survey is Brandt's vole specific remains to be further studied (23).

## Conclusion

In summary, this study first described the prevalence of *Cryptosporidium* spp. in Brandt's vole worldwide. Four *Cryptosporidium* species/genotypes, including a known zoonotic species, were identified in the study area, implicating Brandt's vole could be a potential source of human *Cryptosporidium* infection. Further studies focusing on more host (herdsman, cattle, sheep etc.) as well as source of water to evaluate the transmission network of *Cryptosporidium* spp., especially zoonotic species, in this pastoral area is needed.

## Data Availability Statement

The nucleotide sequences generated in the present study have been deposited in GenBank under accession numbers MT108810 - MT108826.

## Ethics Statement

The animal study was reviewed and approved by the Animal Ethics Committee of the Institute of Zoology, Chinese Academy of Sciences.

## Author Contributions

HH and SF designed the experiments. SF collected the samples. HC, YW, CH, and SH performed the DNA extraction and PCR. SF and HC analyzed the data. SF wrote the manuscript. HH revised the manuscript. All authors contributed to the article and approved the submitted version.

## Conflict of Interest

The authors declare that the research was conducted in the absence of any commercial or financial relationships that could be construed as a potential conflict of interest.
